# Antileukemic Effect of Tualang Honey on Acute and Chronic Leukemia Cell Lines

**DOI:** 10.1155/2015/307094

**Published:** 2015-11-03

**Authors:** Nik Muhd Khuzaimi Nik Man, Rosline Hassan, Cheng Yong Ang, Abu Dzarr Abdullah, Muhammad Amiro Rasheeq Mohd Radzi, Siti Amrah Sulaiman

**Affiliations:** ^1^Department of Hematology, School of Medical Sciences, Universiti Sains Malaysia, 16150 Kubang Kerian, Kelantan, Malaysia; ^2^Department of Medicine, School of Medical Sciences, Universiti Sains Malaysia, 16150 Kubang Kerian, Kelantan, Malaysia; ^3^Department of Pharmacology, School of Medical Sciences, Universiti Sains Malaysia, 16150 Kubang Kerian, Kelantan, Malaysia

## Abstract

Complementary medicine using natural product as antitumor is on the rise. Much research has been performed on Tualang Honey and it was shown to have therapeutic potential in wound healing, and antimicrobial activity and be antiproliferative against several cancer models such as human osteosarcoma (HOS), human breast (MCF-7 and MDA-MB-231), and cervical (HeLa) cancer cell lines. To date, there was limited study on antileukemic properties of Tualang (*Koompassia excelsa*) Honey. The aim of this study was to evaluate the antileukemic effect of Tualang Honey on acute and chronic leukemia cell lines. Leukemia cell lines (K562 and MV4-11) and human mononuclear cell isolated from peripheral blood were grown in RPM1 1640 culture medium. The cells were incubated with increasing concentrations of Tualang Honey. After incubation, the evaluation of viability and apoptosis was performed. The morphological changes of leukemia cells were the presence of cytoplasmic blebs followed by apoptotic bodies and round shape of cells. IC_50_ against K562 and MV4-11 was determined. Tualang Honey gave 53.9% and 50.6% apoptosis activity on K562 and MV4-11, respectively, while on human mononuclear cell it was 37.4%. Tualang Honey has the apoptosis-inducing ability for acute and chronic myeloid leukemia (K562 and MV4-11) cell lines.

## 1. Introduction

Leukemia is malignant neoplasm of white blood cells originating from the bone marrow. It is characterized by the uncontrolled growth of blood cells. Leukemia is classified into acute and chronic leukemia. Chronic myeloid leukemia (CML) and acute myeloid leukemia (AML) develop from the progenitor of myeloid or granulocytic cells. It accounts for nearly 14.6% of all cases of leukemia in Malaysia [1]. The current mode of leukemia treatment is using chemotherapy. However, chemotherapy has its own side effects. The first-line chemotherapy used to treat CML is imatinib mesylate, a tyrosine kinase inhibitor. However it is costly and about 50% developed disease resistance later after treatment [2].

Honey has been used since ancient times for its nutritional and therapeutic value. Many types of honey are available worldwide and its constituents differ from one place to another. Tualang Honey (TH) is a local Malaysian honey, collected from wild honey bees hives on Tualang trees found in the rain forest. It has been used as a complement to treat various diseases where its therapeutic value may be related to its antioxidant properties [3]. In addition, TH also has been shown to have antibacterial, anticarcinogenic, and anti-inflammatory activities.

As an antioxidant, it has several preventative effects against different diseases, such as cancer, coronary diseases, inflammatory disorders, neurological degeneration, and aging. Increase in phenolic compound in TH may contribute to its antioxidant property [4].

Previous study reported that galangin and kaempferol showed their antiproliferative effect on promyelocytic leukemia cells line (HL60) [5, 6]. The substances such as polyphenols and phenolic acids found in honey vary according to the geographical and climatic condition; for example, flavanol kaempferol can be found in rosemary honey and quercetin in sunflower honey. Other study suggested that quercetin was able to induce apoptosis in leukemia K562 cell [7].

To the best of our knowledge there is limited study on the antileukemic effect of TH; thus our aim was to study its apoptosis activity on acute and chronic leukemia cell lines.

## 2. Material and Methods

### 2.1. Cell Culture

Both acute and chronic human leukemia cell lines (K562 and MV4-11, resp.) were purchased from ATCC, USA. These cell lines K562 originated from patient diagnosed as chronic myeloid leukemia and MV4-11 from an acute myelomonocytic leukemia patient. The base medium for this cell line was RPMI 1640, added with fetal bovine serum to a final concentration of 10%, penicillin-streptomycin (pen-strep) 1%. The cell has been cultured with air humidity, 95%; carbon dioxide (CO_2_) 5%; and consistency of temperature at 37.0°C. Cultures are maintained by the addition or replacement of fresh medium. The number of cells seeded for cultures was 1 × 10^5^ viable cells/mL and subcultured at 1 × 10^6^ cells/mL. The medium was renewed every 2 to 3 days.

### 2.2. Isolation of Mononuclear Cells

Mononuclear cells were grown as primary cell line and function as negative control for this study. The cells were isolated by Ficoll Hypaque technique from 5 mL of normal donor blood. The peripheral blood was layered with the ficoll and centrifuged at room temperature (15–25°C) for 30 minutes at 400 ×g with brake-off (Ficoll Paque Plus Instruction Protocol 2007). The mononuclear cells in the buffy layer were collected and washed with Phosphate Buffer Saline (PBS) before incubation with TH for 24 hours at increasing concentrations. These treated mononuclear cells were stained with Monoclonal Anti-Human CD14 to ensure the purity and percentage of CD 14+ mononuclear cells. The CD14 antigen is strongly expressed on monocytes and macrophage. It is particularly of interest to use monocytes as they represent myeloid precursor.

### 2.3. Cytotoxicity Assay

The aim of this assay was to identify 50% Inhibitory Concentration (IC_50_) of treated cell in this study. The cytotoxicity assay was performed using CytoTox 96 Non-Radioactive Cytotoxicity Assay Kit (Promega, USA). The CytoTox 96 Assay quantitatively measures lactate dehydrogenase (LDH), a stable cytosolic enzyme that is released upon cell lysis. This LDH assay was performed on K562 and MV4-11 cell lines and mononuclear cells which were treated with TH at increasing concentration and time at 0.1%, 0.2%, 0.3%, 0.4%, 0.5%, 0.6%, 0.7%, 0.8%, 0.9%, and 1.0% (v/v) for 12 hours, 18 hours, 24 hours, and 48 hours. After the incubation, the supernatant was collected and then incubated in the dark place for 30 minutes. Finally the absorbent was read by using Microplate Elisa Reader.

### 2.4. Morphologic Studies of Cell Line

Morphologic studies using the normal inverted microscope were carried out to observe the morphologic changes of cell death in K562 cell lines elicited by TH. The stage of morphological changes via apoptosis was captured.

### 2.5. Assessment of Apoptosis

Based on cytotoxicity assay, IC_50_ was identified at 24 hours. Harvested K562 and MV4-11 cell lines and mononuclear cells were incubated in 95% air humidity; carbon dioxide (CO_2_) 5%; and consistency of temperature at 37.0°C Then they were incubated with increased concentrations of Tualang Honey for 24 hours. After incubation, the cells were processed using Annexin V Apoptosis Detection Kit. The Apoptosis kit contained Annexin V-FITC and Propidium Iodide (PE) (BD Biosciences, San Jose, CA).

At each identified concentration of TH and at different incubation times, 1 × 10^5^ cells from culture medium were isolated, washed with ice cold phosphate buffer solution, resuspended in 100 *μ*L binding buffer, and stained with 5 *μ*L of FITC-conjugated Annexin V (10 mg/mL) and 10 *μ*L of PI (50 mg/mL). The cells were incubated for 15 minutes at room temperature in the dark, followed by the additional of 400 *μ*L of binding buffer.

Finally the cells were analyzed by a FACScan flow cytometry (Becton-Dickinson, Franklin Lakes, NJ USA). For analysis, K562 and MV4-11 cell lines were gated according to their granularity and size on forward scatter versus side scatter plot. Early apoptosis and late apoptosis were evaluated on fluorescence 2 (FL2 for propidium iodide) versus fluorescence 1 (FL1 for Annexin) plots. The percentage of cells stained with Annexin V only was evaluated as early apoptosis, while the percentage of cells stained with both Annexin V and propidium iodide were evaluated as late apoptosis or necrotic stage.

### 2.6. Statistical Analysis

Data were obtained from at least three independent experiments. The significance of difference was evaluated using Wilcoxon signed-rank test. A probability level of *p* < 0.05 was considered as statistically significant.

## 3. Result

### 3.1. Morphologic Changes by Light Microscopy (Figures 1 and 2)

Morphologically K562 and MV4-11 cell lines were homogenous, oval mononuclear cells. After incubation and treatment with TH, their morphological appearance showed more rounded cells' shape, membrane blebbing, formation of apoptotic body, and finally fragmentation of the cells. These changes correspond to the apoptotic activity.

### 3.2. Effect of Tualang Honey on Cell Viability

**Figure 3:**
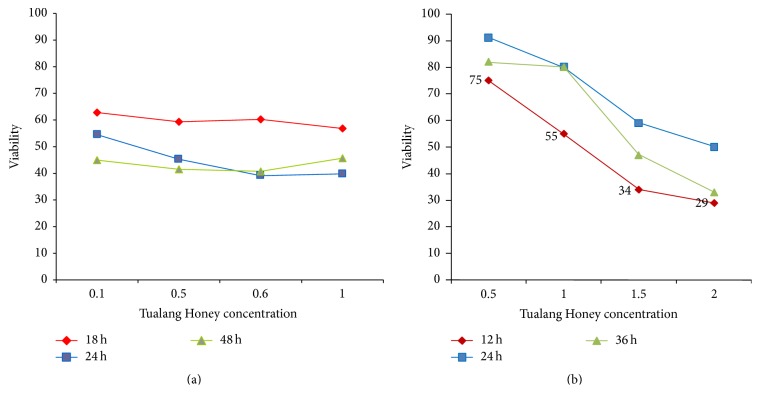
Viability of K562 and MV4-11 cell lines after treatment with Tualang Honey at various concentrations and time points. (a) IC_50_ for K562 cell line was at 0.6% concentration of honey after 24 hours of incubation. (b) IC_50_ for MV4-11 cell line at 12 hours with concentration of 1.2%.

The impact of honey on the cell viability was analyzed by cytotoxicity assay. Exposure of the K562 and MV4-11 cells and mononuclear cells at different concentrations with time-dependent manner showed significant increase in the production of lactate dehydrogenase (Figure 3). The dose inducing 50% (IC_50_) cell growth against normal human mononuclear cells and K562 cell line were determined at 0.6% (18 *μ*L) after 24 hours and MV4-11 leukemia was determined at 1.2% (33 *μ*L) after 12 hours.

### 3.3. Assessment of Apoptosis Activity

**Figure 4:**
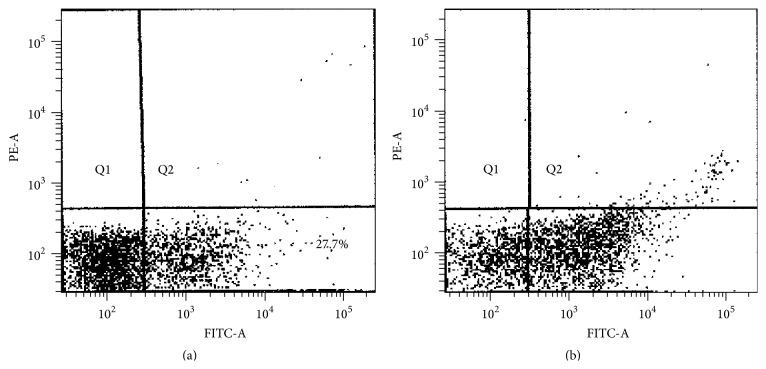
Flow cytometry analysis. (a) Untreated mononuclear cell (control) stained with Mononuclear Anti-Human CD14. (b) Treated mononuclear cell with 0.6% of Tualang Honey. Q4 is a region representing cells undergoing apoptosis while Q3 contains viable cells.

**Figure 5:**
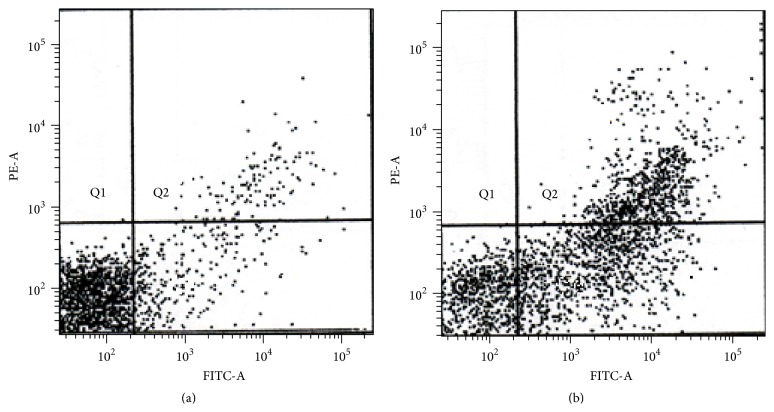
Flow cytometry analysis. (a) Untreated K562 cell line. Viable cells are 86.8%. (b) Treated K562 cell line with 0.6% of Tualang Honey. Viable cells are 38.2%. Q2 and Q4 represent cells undergoing apoptosis and stained with Propidium Iodide and Annexin while Q3 contains viable cells.

**Figure 6:**
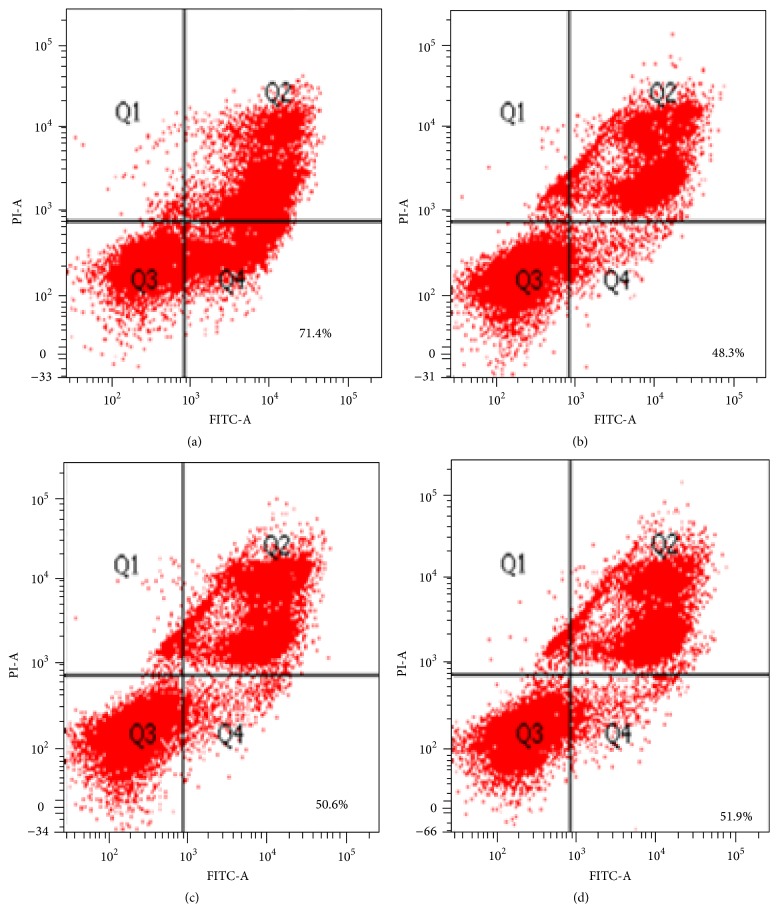
Flow cytometry analysis. (a) Treated MV4-11 cell line with camptothecin as a control. (b) Treated MV4-11 cell line with 1.1% (33 *μ*L) of Tualang Honey. (c) Treated MV4-11 cell line with 1.2% (36 *μ*L) of Tualang Honey. (d) Treated MV4-11 cell line with 1.3% (39 *μ*L) of Tualang Honey. (a), (b), (c), and (d) have been treated for 12 h. Q2 and Q4 represent cells undergoing apoptosis and stained with Propidium Iodide and Annexin while Q3 contains viable cells and stained with Propidium Iodide and Annexin V.

TH was used to determine the apoptosis activity for two leukemic lines and a normal human peripheral blood mononuclear cell. Human peripheral blood mononuclear cell was used as a control. The percentage of apoptosis measured before and after supplement of TH on the normal human peripheral blood mononuclear cell was 27.7% and 37.4%, respectively (*p* > 0.05) (Figure 4). However percentage of apoptosis after treatment with TH at concentration of 0.6% for leukemia cell line K562 (53.9%) was significantly higher than normal mononuclear cells (37.49%) (*p* < 0.05). On the other hand, there was marked reduction in the number of viable cells between untreated (86.8%) and treated K562 (38.2%) cell lines (*p* < 0.05) (Figure 5). Similarly for MV4-11 cell line, the apoptosis activity showed dose-dependent manner (Figure 6). At concentrations of 1.1%, 1.2%, and 1.3% of TH, the percentage of apoptosis was 48.3%, 50.6%, and 51.9%, respectively. Quantitative analysis using Annexin V/PI assay showed increase in both early and late apoptosis of K562 and MV4-11 cell lines after treatment with TH.

## 4. Discussion

In Southeast Asia, three types of honey bees can be found in farm, forest, and villages. The type of honey that was used in our study was TH which has been produced by the forest bees on the branches of the Tualang tree, Asia's tallest tree (growing up to 80 meters high). The Tualang tree grows in the lowland rainforests of southern Thailand, Peninsular Malaysia, Northeastern Sumatra, Borneo, and Palawan. Physicochemical properties of TH are dark brown appearance; pH was 3.55 and specific gravity 1.335. TH contains higher content of phenolic acids, flavonoids, and 5-(hydroxymethyl) furfural (HMF) than other local Malaysian honeys. A total of six phenolic acids (gallic, syringic, benzoic, transcinnamic, p-coumaric, and caffeic acids) and five flavonoids (catechin, kaempferol, naringenin, luteolin, and apigenin) are found in TH [4]. Hydrocarbons constitute more than half (58.5%) of its composition. These include alcohols, ketones, aldehydes, furans, terpenes, flavonoids, and phenols. Some compounds found in TH previously not reported in other honeys are stearic acids, 2-cyclopentene-1,4,-dione, 2[3H]-furanone or dihydro-butyrolactone, gamma-crotonolactone or 2[5H]-furanone, 2-hydroxy-2-cyclopenten-1-one, hyacinthine, 2,4-dihydroxy-2,5-dimethyl-3[2H]-furan-3-one, and phenylethanol [8].

To date there is limited study on the effect of honey on leukemia cell lines. Most studies using honey were performed on solid cancers and showed anticancer properties. In this study, the cytotoxic and apoptotic effects of honey on K562 and MV4-11 leukemia cell line were investigated. K562 is a chronic myeloid leukemia cell line with a molecular phenotype bcr/abl gene fusion transcript while MV4-11 is a myelomonocytic leukemia having inversion 16 karyotype. The ability to induce cell apoptosis is an important property for antileukemic effect. Cell death or apoptosis can be induced by any highly concentrated materials through the mechanism of osmosis by making the cell membrane permeable to water and causing cancer cells to swell and die. TH has high sugar content of 30% and produces an osmosis effect [9]. In order to provide evidence and control the condition, blood monocytes and macrophage from normal human mononuclear cells were isolated and incorporated into the study as control cells. This study has proved that TH did not significantly cause apoptosis on the normal cells as activity before and after supplement of TH was 27.7% and 37.4%, respectively (*p* > 0.05%).

On the other hand, there was a significant difference on the percentage of apoptosis between K562, MV4-11, and normal mononuclear cells with activity of 53.9%, 50.6%, and 37.4%, respectively (*p* < 0.05), using TH. It can be concluded that TH has greater potential as apoptosis induces agent for myeloid leukemia cells.

Apoptosis is characterized by distinct morphologic features, including chromatic condensation, cell and nuclear shrinkage, membrane blebbing, and oligonucleosomal DNA fragmentation. The apoptosis was confirmed by Annexin V (apoptosis detection kit) after exposure to TH. In this study the apoptosis of leukemia cell lines was prominent during the late stage exhibited by double positive cells by Annexin/PI in comparison to normal human mononuclear cell, where the apoptosis was at the early stage.

The apoptosis activity could be attributed by few possible compounds such as flavonoids. One of its chemical structures, namely, quercetin, has been reported to inhibit pancreatic and breast cancer cell growth and induce apoptosis via Bcl-2 expression downregulation and upregulation of Bax expression [10, 11].

Myeloid leukemia either AML or CML is a group of malignant disorders characterized by uncontrolled proliferation of clonal hematopoietic precursor cells associated with impairment of normal hematopoiesis. Approximately 50–75% of adult patients with AML achieve complete remission when treated with cytarabine and daunorubicin or idarubicin [12]. This chemotherapy induces cell death by apoptosis mainly mediated through the intrinsic mitochondrial pathway but also through the extrinsic death receptor pathway, both of which lead to caspase activation and cell death. The mode of action by caspases either acts as initiator caspases (which include caspases 8, 2, 9, and 10) or effector caspases (caspases 3, 6, and 7). The initiator acts at the early phase of apoptotic process, while the effector acts at the late stage.

The role of apoptosis in the pathogenesis of AML has been elucidated over the past years. Some fusion proteins interact with mediators of apoptosis, sending antiapoptotic signals that favor the growth of leukemic cells: PML/RAR-*α* through the p53 pathway or AML1/ETO through the Bcl2-related pathway. These mediators are responsible towards chemoresistance and as such new compounds are required to induce the apoptosis of leukemic cells. The treatment strategy of any new compound aims to selectively target and kill the leukemia cells with no or limited collateral damage to normal hematopoietic progenitor cells.

Our study has shown that TH has the capacity to induce apoptosis of both myeloid leukemia cell lines with minimal effect on the normal mononuclear cells.

Interestingly TH induced a greater extend of apoptosis in acute myeloid leukemia (MV4-II) compared to chronic myeloid leukemia (K562). Future study is required to understand the mechanism of apoptosis underlying both types of myeloid leukemia. As natural product, TH has a great potential to serve as antileukemic agents.

## 5. Conclusion

This study has proved that TH did produce a significant apoptosis effect on both acute and chronic leukemia cell lines. Thus we conclude that TH could potentially serve as an antileukemic agent with the apoptosis properties.

## Figures and Tables

**Figure 1:**
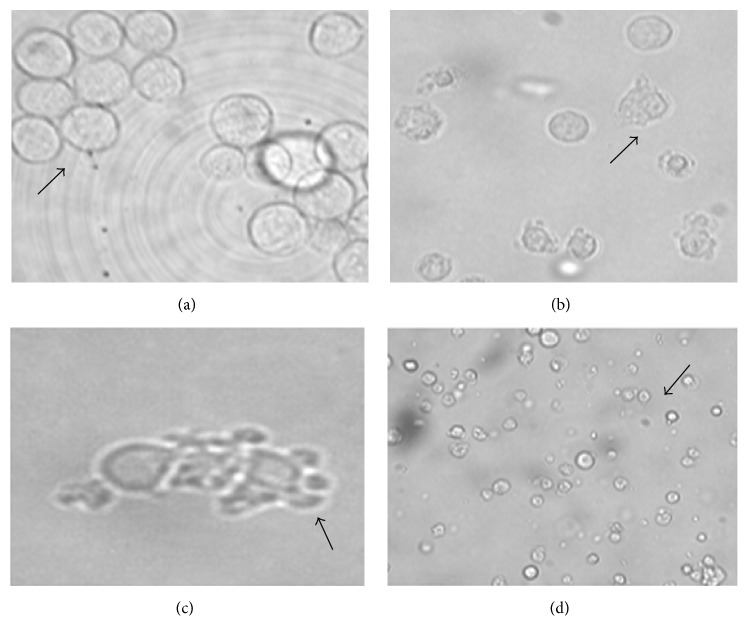
Photomicrograph of K562 cell line treated with 0.6% (18 *μ*L) Tualang Honey at 24 hours. Arrow marks indicate morphology of K562 cell line and stage of apoptosis. (a) Normal morphology of K562 cell line. (b) Treated K562 cell line shows the membrane blebbing as activity of early apoptosis. (c) Formation of apoptotic body. (d) Cell lysis: the final stage of apoptosis.

**Figure 2:**
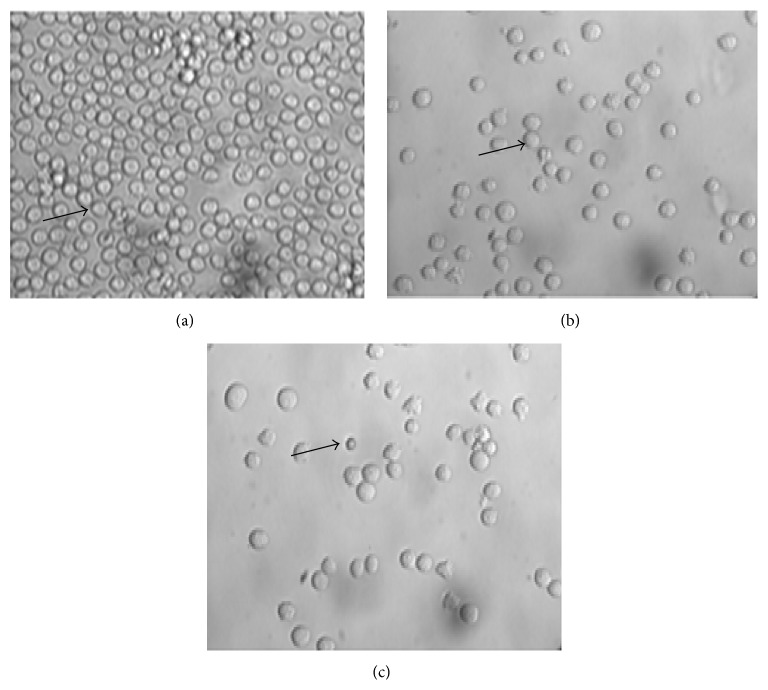
Photomicrograph of MV4-II cell line treated with 1.2% (33 *μ*L) Tualang Honey at 12 hours. Arrow marks indicate morphology of MV4-II cell line and stage of apoptosis. (a) Normal morphology MV4-II cell line. (b) Treated MV4-II cell line shows the membrane blebbing as activity of early apoptosis. (c) Formation of apoptotic body.
